# Substantial Genetic Differentiation Within and Between Populations of the European Adder (*Vipera berus*) in Baden‐Württemberg, Germany

**DOI:** 10.1002/ece3.71644

**Published:** 2025-07-14

**Authors:** P. Lennart Schmid, Judith Grünewald, Marc I. Förschler, Eva Maria Griebeler

**Affiliations:** ^1^ Institute of Organismic and Molecular Evolutionary Biology Johannes‐Gutenberg University Mainz Mainz Germany; ^2^ Department for Ecological Monitoring, Research and Species Protection Black Forest National Park Seebach Germany

**Keywords:** conservation, genetic diversity, microsatellites, population genetic structure, reptiles, snakes

## Abstract

Habitat destruction and fragmentation are the main threats to species' long‐term survival, they isolate populations geographically and genetically. Over the last centuries, the European adder ( *Vipera berus* ) has experienced a decline in abundance and an increase in population extinctions within its entire distribution area. In our study, we aimed to infer whether these trends that are also observed in Baden‐Württemberg, Germany have affected the genetic constitution of adders in this region. We therefore genotyped 141 adders using 10 microsatellite markers. Adders were sampled at eight sites, six in the northern Black Forest, one in the southern Black Forest and one in the Swabian Jura. We analyzed genetic diversity, genetic differentiation within sites, average pairwise relatedness and sibling relationships at each site. We additionally applied population STRUCTURE analyses on all and to a subset of individuals and we assessed genetic differentiation between sites. Genetic diversities inferred were like those found in other European regions. We detected signs of genetic erosion across all sites, that is, an excess of homozygotes, positive *F*_IS_ values, large mean pairwise relatedness values and/or the presence of full and half sib dyads. At one site, we found clear evidence for a within‐site differentiation by STRUCTURE and sibship clustering, which we attribute to a barrier to gene flow, that is, a road bisecting the site. We inferred high genetic differentiation between all sites, indicating low gene flow between sites. Our findings indicate that conservation measures should increase population sizes and restore gene flow within and between adder populations in Baden‐Württemberg.

## Graphical abstract

The number of records of the European adder *Vipera berus* has declined substantially during the last decades in Baden‐Württemberg, Germany from habitat fragmentation and destruction. We genotyped 141 individuals sampled at eight sites in this region and evaluated 10 microsatellite markers. We found high average pairwise relatedness values and close sibling relationships for all sites, indicating high genetic differentiation within sites. Differentiation between sites was also high. In summary, our results indicate genetic erosion and a low gene flow within and between sites and ask for conservation measures to enable long‐term survival of the adder in Baden‐Württemberg.

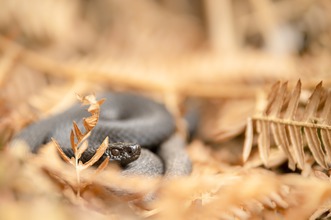

## 1 Introduction

Reptiles are the most species‐rich group among terrestrial vertebrates (Tingley et al. 2016). The majority of terrestrial reptiles have low dispersal abilities and habitat loss has been proven as the largest current threat to their extinction (Alford and Richards 1999; Gardner et al. 2007; Gibbons et al. 2000). In particular, cold‐adapted reptile species often suffer from urbanization, agriculture and forestry (Gardner et al. 2007; Laufer et al. 2007; Otte et al. 2020). Conversely to their global extinction risk (IUCN 2025), in Germany, reptiles comprise the highest proportion of species threatened to extinction across all vertebrate groups (Aßmann and Finck 2020).

**FIGURE 1:**
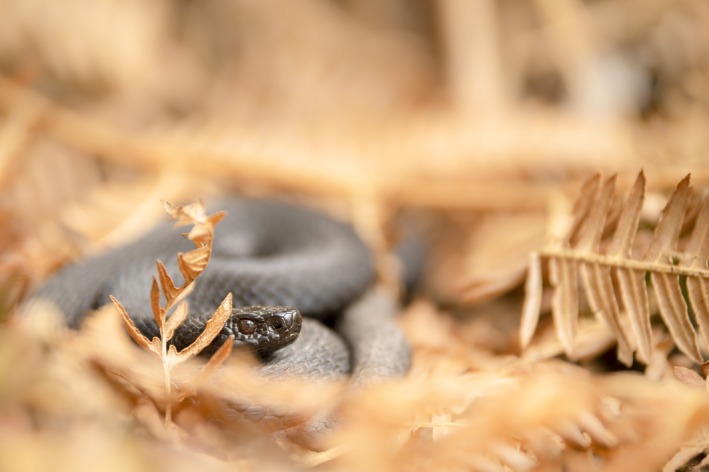
Melanistic adult male of *Vipera berus* sampled at the site NP3. Picture by P. Lennart Schmid.

The European adder *Vipera berus* (Linnaeus 1758) (Figure 1) is a cold‐adapted snake with a large boreal distribution. In Germany, it is found in several mountainous regions in the south (Swabian Jura, Bavarian Forest, Alps and foothills of the Alps), in low mountain ranges of the east and in the lowlands in the north (Völkl and Thiesmeier 2002). It primarily inhabits open landscapes such as heathlands, bogs, and fens. The current IUCN Red List status (2025) of the adder is “least concern,” which seems to be justified by its wide distribution, its tolerance of a broad range of habitats, and a presumed large population. However, this status contradicts that populations are declining in many regions within its entire distribution area (Völkl and Thiesmeier 2002) and in particular that global warming might have a negative impact on the survival of the cold‐adapted adder (Guillon 2012). For example, in Germany, adder populations have generally experienced a decline in size of 50%–70% during the last century (Völkl and Thiesmeier 2002).

Not only in Germany (Otte et al. 2020), but also in most western European countries, the venomous adder has undergone considerable persecution during the 19th and 20th centuries (Ursenbacher et al. 2009; Völkl and Thiesmeier 2002). This resulted in declining populations and even eradication of populations. Adders are viviparous, have a low vagility with a sex‐based dispersal in favor of males, are highly philopatric and polyandrous females show low annual fecundities (Bauwens and Claus 2019; François et al. 2021; Luiselli 1993; Madsen and Shine 1992a, 1992b). Such a combination of life history traits hampers re‐establishment of populations after a substantial reduction in size as well as an establishment of new populations and exchange of individuals between populations. Given that in small populations mating opportunities are limited, it can also promote a decrease in genetic diversity and an increase in the risk of inbreeding depression (Gardner et al. 2019; Madsen et al. 1996). Madsen et al. (1996) observed a high relatedness between individuals, small litter sizes and high proportions of deformed and stillborn offspring for a small and isolated adder population in Sweden due to mating of closely related individuals and inbreeding depression. Likewise, Ball et al. (2024) reported the finding of a clutch of five dead newborn adders in a nature reserve in eastern England for the year 2015. This population was founded by a translocation of seven adders in 1999 and seemed to thrive.

In changing environments, for example, due to anthropogenic climate change, populations with a high genetic diversity will more likely adapt to altered conditions than populations of a low diversity (Frankham 1996). So far, few studies have addressed the genetic diversity of adder populations across Europe (Ball et al. 2020, 2024; Bauwens et al. 2018; Madsen et al. 1996; Pozzi et al. 2023; Ursenbacher et al. 2009; Ursenbacher et al. 2015). In particular, in Germany, only one population in the south of the Black Forest, Baden‐Württemberg (Southern Germany) has been studied. It was included in a study of Ursenbacher et al. (2015) on the postglacial recolonization of Western Europe by the adder, in which the authors applied microsatellite analysis to 32 populations. This Black Forest population (*N* = 21) not only had the lowest allelic richness (1.35) and expected heterozygosity (0.09) when compared to the five other Jura Mountain populations (1.85–3.32, 0.29–0.54), but also of all 32 populations studied by these authors. Ursenbacher et al. (2015) hypothesize that its exceptionally low genetic diversity was caused by a bottleneck or a founder effect in the past. Their interpretation is consistent with the substantial persecution of adders during the 19th and 20th centuries (Otte et al. 2020) as well as with ongoing habitat fragmentation and loss from scrub encroachment, forestation, and urbanization (Fritz et al. 2007) in this region. For example, the Zweribach region located in the south of the Black Forest lost about 60% of open landscapes between 1800 and 2000 (Ludemann 2015).

As persecution (Ursenbacher et al. 2009; Völkl and Thiesmeier 2002) and changes in land use (Fritz et al. 2007) led to a substantial decline and fragmentation of adder populations in Baden‐Württemberg, we investigated the genetic differentiation within and between adder populations in this region. Our results will provide new insights needed for the conservation of the adder in Baden‐Württemberg and in other mountainous regions in southern and eastern Germany. They will also support current conservation strategies on adder populations in the Black Forest.

**FIGURE 2:**
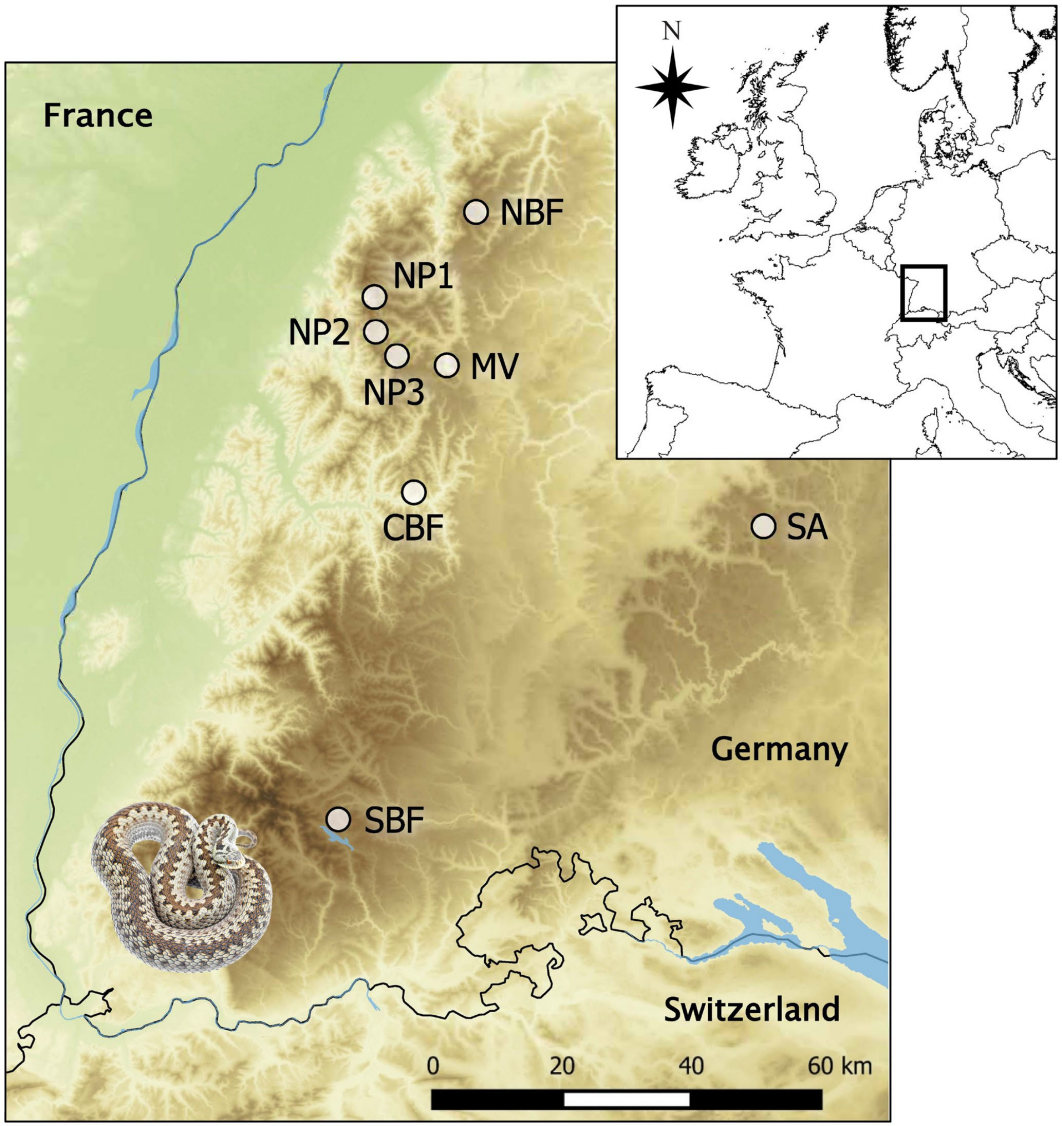
Locations of the eight sampling sites (NBF, NP1, NP2, NP3, MV, CBF, SBF, SA) of *Vipera berus* in the Black Forest and the Swabian Jura in Germany. For the protection of the species, exact geographic locations of sites are available only from the authors PLS and JG.

To this end, we genotyped a total of 141 *V. berus* individuals (131 from the Black Forest and 10 from the Swabian Jura, Baden‐Württemberg) and evaluated 10 microsatellite markers of which six were developed for *V. berus* (Carlsson et al. 2003; Ursenbacher et al. 2009), three for *Vipera ursinii* (Metzger et al. 2011) and one for *Vipera aspis* (Geser et al. 2013). Individuals were sampled at six sites in the northern Black Forest, at one site in the southern Black Forest and at one site in the Swabian Jura (Figure 2).

## 2 Materials and Methods

### 2.1 Study Sites and Sample Sizes

We sampled 134 adder individuals across eight sites in Baden‐Württemberg (BW), Southern Germany between March and October 2021. Selection of sampling sites was based on distribution data of adders in BW, which suggested occurrence gaps between selected sites (Fritz et al. 2007).

For the protection of the endangered adder, we only use abbreviations on our sampling sites in this paper. Six of the sites are in the northern Black Forest (NBF, NP1, NP2, NP3, MV, CBF), one in the southern Black Forest (SBF), and one in the Swabian Jura (SA) (Figure 2). To investigate genetic differentiation of adders within and between sampling sites at smaller and larger geographic scales in BW, we chose six sites in the northern Black Forest that are much more closely located to each other than the two others (Figure 2). The largest site sampled was SBF (ca. 24.89 ha) followed in descending order by NP1 (ca. 22.88 ha), NP2 (ca. 19.74 ha), NBF (ca. 17.64 ha), NP3 (ca. 12.93 ha), CBF (ca. 11.85 ha), and MV (ca. 5.84 ha) and SA was the smallest (ca. 4.24 ha). Each of these area sizes was estimated in QGIS (Version 3.10.4) from aerial images (taken from Google Earth) and equals the total sum of areas covered by open habitats and located within the minimum convex polygon defined by the site's adder records. Five sites, at which we sampled individuals, were natural habitats, such as heaths, bogs, and open forests, that is, NBF (*N* = 15), NP1 (*N* = 15), NP2 (*N* = 20), NP3 (*N* = 29), and SBF (*N* = 19). The other three sites sampled, that is, MV (*N* = 12), CBF (*N* = 14), and SA (*N* = 10) are shaped substantially by anthropogenic structures such as rock walls, railways, and cropland. One of the sites classified as natural habitat (NP3) is crossed by a highly frequented road. For additional analysis on NP3, we were lucky to sample four individuals in between the sites NP1 and NP2 and four others in a village south of NP3 in October 2021, where adders are rarely observed. The site CBF is transected by a village.

Our sampling period (March–October) covered the entire activity period of the adder in BW. To maximize sample sizes and to ensure a fairly even sampling of males and females, we sampled each site three times in March/April, once in June, August, and September/October, respectively. This resulted in six sampling days per site at which we searched each area entirely for adders. In the months of March and October, sampling was conducted on days with a medium to high insolation and in the months of April through September on days with a medium to low insolation, low wind velocity, and air temperature between 5°C and 20°C. For none of the sites were we able to sample adders at all 6 days (NBF: 0/5/2/3/5/0; NP1: 0/0/2/4/8/1; NP2: 7/4/1/0/4/3; NP3: 0/13/9/0/0/7; MV: 0/0/0/12/0/0; CBF: 3/2/0/0/9/0; SBF: 0/0/9/0/10/0; SA: 0/5/0/0/0/5; sequences give numbers of adders sampled at the 6 days). Male to female ratios of sampled individuals differed among sites (NBF: 8/7; NP1: 5/10, NP2: 12/7; NP3: 12/15; MV: 3/9; CBF: 5/9; SBF: 14/5; SA: 6/4) and were biased for most sites. To avoid multiple sampling of individuals, pictures of their head scales were taken. Hereafter, the term population is used for all adder individuals that were sampled at a site throughout the sampling period.

### 2.2 Sampling of Adder Individuals and DNA Extraction

All samples of adders were obtained from buccal swabs containing DNA from mucosal epithelial cells. After sampling, all snakes were released exactly where they had been found. Buccal swabs were transferred into Eppendorf tubes containing 70% ethanol in the field. Tubes were stored at −20°C in the lab until DNA extraction was conducted.

The DNA was extracted from the mucosal epithelial cells by applying an adjusted Epicenter Master Pure gDNA protocol to achieve sufficiently high DNA concentrations for microsatellite amplification. We made three adjustments to this protocol. (1) We dried the buccal swabs (i.e., the cotton ball with mucosal epithelial cells) instead of homogenizing a tissue sample. (2) After adding proteinase K to the solution, we additionally incubated the samples for 30 min at 63°C. (3) After having conducted step eight of the manufacturer's protocol, we removed the cotton ball from the solution. Finally, the DNA concentration of each solution was measured with a Peqlab Nanodrop 1000 spectrophotometer, and solutions of concentrations exceeding 60 ng/μL were diluted with Low TE to a maximum concentration of 30 ng/μL. The buccal swab yielded no DNA (DNA yield = 0.00 ng/μL) for one of the individuals sampled at NP2 (*N* = 20). Thus, the final number of genotyped individuals of NP2 was 19. For more information on DNA yields per swab, refer to Table A1 in Appendix.

### 2.3 Microsatellite Amplification and Preparation

We amplified 13 microsatellite loci that were established for the *Vipera* genus to genotype each of the 139 *V. berus* individuals. The primers Vb‐A8, Vb‐A11, Vb‐B'2, Vb‐B10, Vb‐B'10, Vb‐D'10 (Ursenbacher et al. 2009) as well as Vb3 and Vb21 (Carlsson et al. 2003) were developed for *V. berus* , whereas Vu16, Vu29, Vu32, and Vu38 (Metzger et al. 2011) were developed for *V. ursinii* and Va08 (Geser et al. 2013) for *V. aspis* (Table 1). Multiplexing was successful for three primer pairs: Vb‐B'2 and Vb‐B'10, Vb‐A11 and Vb‐D'10, Vb‐A8 and Vb‐B10 (Table 1).

**TABLE 1:** The 13 microsatellite loci applied to *Vipera berus* in this study.

Species	Locus	Primer sequence (5′–3′), forward and backward	Repeat motif	Annealing temp. (°C)	Source
*V. berus*	Vb‐A8	F: ATTTCACCATGCCTCCAGAA B: GGTACACTCATTGTGATGAAC	CA	55	Ursenbacher et al. (2009)
*V. berus*	Vb‐B10	F: CGTGAGGTGTGTAAAATGAAG B: CTATTTGAATCCCACCAGTG	GA	55	Ursenbacher et al. (2009)
*V. berus*	Vb‐A11	F: GGTCAGTAACATCAGTCTGC B: AGTCTTCCCTTACTTTGGCG	CA	58	Ursenbacher et al. (2009)
*V. berus*	Vb‐D'10	F: GTCCTCCTTATCATCTATCC B: CCTGGGTGCTCTCTCAG	AAG	58	Ursenbacher et al. (2009)
*V. berus*	Vb‐B'2	F: CTGAACAGAACAGGAGGAG B: GGAAAACGAAGCAGTCAGGC	GA	58	Ursenbacher et al. (2009)
*V. berus*	Vb‐B'10	F: GCAAATTATTTTTGGGGTAGG B: GAGATAGAGAACAAGTAGAGG	GA	58	Ursenbacher et al. (2009)
*V. berus*	Vb3	F: CAAGAAATGGAGATGAGC B: GAAACCTATGAGCCAGTA	AC	52	Carlsson et al. (2003)
*V. berus*	Vb21	F: CCAGTGGCACATAAGTAG B: GTTCCATCATCAAAACAT	TG	53	Carlsson et al. (2003)
*V. ursinii*	Vu16	F: TCATTCTGGCTCTAATACCACATC B: GGAGGAAATGAGAAGACTGGC	ATTT	55	Metzger et al. (2011)
*V. ursinii*	Vu29	F: GTAGGAGTCACTTGCTC B: GTTCATGGTCCTGTCCCTTTGG	CTA	60	Metzger et al. (2011)
*V. ursinii*	Vu32	F: TCTTTGGCTGCTTCAAGATTAG B: GCAGTAAGATGGAATTGG	CA	50	Metzger et al. (2011)
*V. ursinii*	Vu38	F: GGAAATCTGGTTGGAGCCCCC B: GCACTGTCTGTCACACC	AC	60	Metzger et al. (2011)
*V. aspis*	Va08	F: CCCTGATCTCCCTTGTTAATGC B: CTGGACAGCCACTTGTCTG	CAAT	62	Geser et al. (2013)

*Note:* Shown are the *Vipera* species for which each locus was originally developed, locus name, primer sequences (forward and backward), repeat motif, annealing temperature (°C) published and used by us as well as sources of information on loci. Primer pairs with equal annealing temperatures Vb‐A8 and Vb‐B10, Vb‐A11and Vb‐D'10, and Vb‐B'2 and Vb‐B'10 were multiplexed.

We used the QIAGEN multiplex kit for polymerase chain reactions (PCR) with fluorescently labeled primers for each DNA sample and adopted the protocol provided by this manufacturer. We amplified 1.5 μL DNA solution, with 5 μL Qiagen Multiplex Mix, 1 μL Primer‐Mix (2 μmol/L of each primer) and 2.5 μL RNase‐free water, yielding a final solution of a volume of 10 μL. PCRs were implemented on a BIOMETRA Professional Gradient 96 thermocycler. For all loci, the annealing temperatures given in the literature were used (see Carlsson et al. 2003; Geser et al. 2013; Metzger et al. 2011; Ursenbacher et al. 2009). We multiplexed only loci with equal annealing temperatures (Table 1). All PCRs were conducted under the following cycling conditions: 90 s at 97°C for initial denaturation, followed by 35 cycles of 94°C for denaturation (30 s), 52 up to 62°C for annealing depending on the locus (40 s, Table 1), 72°C for elongation (60 s) and 30 min of 72°C for the final elongation. PCR products were stored at 10°C until they were processed at StarSEQ GmbH, Mainz. PCR fragment lengths were run on a 96‐capillary 3730xl DNA Analyzer (ABI 3730). Fragments were analyzed in Geneious 11.0.5 (https://www.geneious.com) using the plugin “microsatellite” provided by the manufacturer of this software. Allele sizes and bins were manually scored, and allele peaks were scored following Arif et al. (2010).

### 2.4 Null Alleles and Linkage Disequilibria

To identify null alleles, stuttering and large allelic dropouts we used the software MicroChecker 2.2.3 (van Oosterhout et al. 2004). Loci with null alleles were removed from the corresponding sites in the dataset. The resulting dataset was uploaded to the web version of GenePop 4.7 (Raymond and Rousset 1995) to assess potential linkage disequilibria between all pairs of loci for each of the eight sites. In this analysis, we used the default settings including those for Markov chain parameter values. Loci showing linkage equilibria were removed either only from the respective sample of the site or when they were significant for at least two sites from the entire dataset.

### 2.5 Genetic Differentiation Within and Between Sites

Exact tests on Hardy–Weinberg equilibrium, heterozygosity deficiency and excess were performed in Genepop 4.7 (Raymond and Rousset 1995) with all loci and for each site. We further calculated the numbers of effective (*N*_E_ *A*) and of private alleles (*A*_P_) as well as the observed (*H*_O_) and expected heterozygosity (*H*_E_) across loci in GenAlEX 6.503 (Peakall and Smouse 2012). To assess the average relatedness of individuals at each site, we used this software again (*r*, Lynch and Ritland 1999). We also established the average number of alleles standardized to the smallest sample size (allelic richness, *A*_R_) and the coefficient of inbreeding (*F*_IS_) across loci for each site by applying the software FSTAT 2.9.3.2 (Goudet 1995).

As STRUCTURE analysis (Pritchard et al. 2000) suggested two genetically distinct subgroups for site NP3 and CBF, respectively (see Section 3), all these previous analyses on genetic diversity and within genetic differentiation were repeated for each of the two subgroups inferred for NP3 and CBF, respectively.

To quantify genetic differentiation of adders between the eight sampling sites, we calculated pairwise fixation indices (*F*_ST_) in FSTAT. We then established the relationship between pairwise *F*_ST_ values and geographic distance (log_10_[km]) (isolation by distance, Wright 1943) and checked its significance by Mantel tests in R (Version 4.2.2).

For testing the significance of *F*_IS_ and pairwise *F*_ST_ values, we calculated 95% confidence intervals on *F*_IS_ and pairwise *F*_ST_ values in R (Version 4.2.2) using the “hierfstat” package and 10,000 permutations. The 95% confidence intervals on *N*_E_ *A*, *A*_R_, *A*_P_, *H*_O_, *H*_E_ and *r* values used to assess differences between sites are outputted by Genepop 4.7 and GenAlEX 6.503, respectively.

Unless otherwise noted, we adopted the default settings when applying all previously mentioned tools.

### 2.6 STRUCTURE Analysis and Sib Ship Analysis in COLONY

We established genetic groups and admixture among groups in the software STRUCTURE 2.3.4 (Pritchard et al. 2000) using all individuals from the eight sites. Therefore, parameter values on the Markov chain Monte Carlo (MCMC) approach were chosen as recommended by Evanno et al. (2005), that is, the burning‐in period was 10,000, K was 15 with 20 runs per K and the optimal number of K was identified from the “delta K” criterion. We visualized the results from STRUCTURE using the “stringr” package in R (Version 4.2.2).

For a better understanding of the genetic differentiation of individuals sampled at NP3, a second STRUCTURE analysis was carried out analogously, but only with the individuals from NP1, NP2, and NP3, and the eight individuals sampled in the vicinity of these sites in October 2021.

As NP3 and CBF showed several signs on an excess of homozygotes (see Section 3), we explored full‐ and half‐sib dyads for each of the eight sites, using the software COLONY 2.0.6.7 with standard settings (Jones and Wang 2010).

### 2.7 BOTTLENECK Analysis

By applying the software BOTTLENECK 1.2.02 and using the infinite‐allele mutation model and standard settings (Cornuet and Luikart 1996), we assessed a past reduction in effective population size for each of the eight sites from a bottleneck (i.e., a drastic, temporary reduction in population size) or a founder effect due to persecution of adders (Schiemenz 1985), changes in land use (Fritz et al. 2007; Ludemann 2015), or historic postglacial recolonization (Ursenbacher et al. 2015).

## 3 Results

### 3.1 Null Alleles and Linkage Disequilibrium

We started with 13 different microsatellite loci to genotype adder individuals (Table 1). The markers Vu38 and Vb21 turned out to be monomorphic for an initially tested subset of 32 individuals, which could indicate allelic dropouts for both loci. The other 11 markers did not show any signs of stuttering or allelic dropouts, except for NP3. For NP3, null alleles were indicated for marker Vb‐A8 and evident for two individuals, but this excess of homozygotes disappeared when analyzing each of the two distinct subgroups that STRUCTURE analysis had inferred for NP3 (see below). As the marker Vb‐A8 showed significant nonrandom associations with two other markers across all sites (Vb3, all *p* values ≤ 0.021 and Vb‐A11, all *p* values < 0.001), we rejected this marker completely. Thus, our final sample size on NP3 was 27, and our final dataset analyzed consisted of 139 individuals (= 131 from the eight sites plus eight in the vicinity of NP3), for which genotypes from the 10 markers that did not show pairwise linkage disequilibria were established.

### 3.2 Genetic Diversity and Genetic Differentiation Within Sites

Out of all eight sites, NP2 had the highest effective number of alleles (*N*_E_ *A* = 3.246, Table 2) and the highest allelic richness (*A*_R_ = 4.147, Table 2), whereas MV had the lowest effective number of alleles (*N*_E_ *A* = 1.945, Table 2) and the lowest allelic richness (*A*_R_ = 2.533, Table 2). Allelic richness did not correlate with sample size (Spearman's rank correlation, *ρ* = −0.143, *p* value = 0.752). We found private alleles at all sites (Table 2), with CBF, NP1 and SA having the most (*P*_A_ = 4) and NBF having the fewest (*P*_A_ = 1) (Table 2).

**TABLE 2:** Summary statistics of individuals of *Vipera berus* sampled at eight sites.

Pop	*N*	HW	*N*_E_ *A*	*A*_R_	*A*_P_	*H*_O_	*H*_E_	*F*_IS_	*r*
NBF	15	0.878	2.468 (2.319, 2.617)	3.258 (2.763, 3.753)	1 (0.494, 1.506)	0.513 (0.484, 0.543)	0.537 (0.508, 0.566)	0.078 (−0.002, 0.165)	0.134 (0.125, 0.160)
NP1	15	0.665	2.491 (2.291, 2.690)	3.473 (2.646, 4.300)	4 (3.174, 4.826)	0.520 (0.491, 0.549)	0.533 (0.508, 0.559)	0.059 (−0.057, 0.166)	0.093 (0.074, 0.116)
NP2	19	0.299	3.246 (2.950, 3.543)	4.147 (3.222, 5.072)	3 (2.313, 3.687)	0.653 (0.631, 0.675)	0.616 (0.595, 0.638)	−0.032 (−0.130, 0.052)	0.051 (0.040, 0.066)
NP3	27	< 0.001	2.967 (2.724, 3.193)	3.710 (2.913, 4.243)	2 (1.497, 2.503)	0.526 (0.501, 0.534)	0.570 (0.551, 0.593)	0.095* (0.030, 0.190)	0.067 (0.041, 0.060)
NP3‐E	13	0.010	2.403 (2.161, 2.622)	3.015 (2.309, 3.721)	1 (0.456, 1.544)	0.400 (0.373, 0.441)	0.485 (0.448, 0.523)	0.213* (0.086, 0.310)	0.077 (0.054, 0.085)
NP3‐S	14	0.076	2.684 (2.486, 2.883)	3.121 (2.544, 3.698)	0 (0.000, 0.000)	0.643 (0.598, 0.642)	0.561 (0.544, 0.596)	−0.05 (−0.151, 0.033)	0.130 (0.098, 0.137)
MV	12	1.000	1.945 (1.845, 2.046)	2.533 (2.029, 3.037)	2 (1.246, 2.754)	0.483 (0.454, 0.513)	0.444 (0.415, 0.474)	−0.044 (−0.112, 0.016)	0.281 (0.259, 0.310)
CBF	14	0.002	2.550 (2.398, 2.701)	3.690 (3.049, 4.331)	4 (3.145, 4.885)	0.493 (0.457, 0.529)	0.544 (0.507, 0.580)	0.130 (−0.007, 0.248)	0.072 (0.043, 0.088)
CBF‐E	6	0.809	1.937 (1.761, 2.113)	2.600 (1.960, 3.240)	1 (0.200, 1.800)	0.417 (0.353, 0.480)	0.408 (0.348, 0.469)	0.071 (−0.125, 0.219)	0.220 (0.139, 0.270)
CBF‐W	8	0.005	2.485 (2.256, 2.714)	3.342 (2.474, 4.210)	1 (0.307, 1.693)	0.550 (0.494, 0.605)	0.525 (0.477, 0.573)	0.019 (−0.114, 0.158)	0.079 (0.047, 0.107)
SBF	19	0.257	2.252 (2.121, 2.383)	3.388 (2.752, 4.042)	3 (2.313, 3.687)	0.526 (0.497, 0.554)	0.497 (0.472, 0.522)	−0.028 (−0.058, 0.092)	0.184 (0.180, 0.208)
SA	10	0.374	2.447 (2.275, 2.619)	3.638 (3.044, 4.232)	4 (0.299, 0.501)	0.467 (0.436, 0.498)	0.538 (0.504, 0.572)	0.185* (0.133, 0.276)	0.123 (0.095, 0.162)

*Note:* Ten microsatellite loci were evaluated. STRUCTURE analysis (Figure 3A) suggested that NP3 and CBF each consists of two subgroups (NP3‐E & NP3‐S and CBF‐E & CBF‐W). 95% confidence intervals in brackets.

Abbreviations: *A*_P_ = number of private alleles; *A*_R_ = allelic richness; *F*_IS_ = inbreeding coefficient (significant values are marked by an asterisk); *H*_E_ = expected heterozygosity; *H*_O_ = observed heterozygosity; HW = Hardy–Weinberg equilibrium (*p* value); *N* = number of individuals genotyped; *N*_E_ *A* = number of effective alleles; *r* = mean pairwise relatedness of individuals within a site. * Significant deviation from zero.

Observed heterozygosities (*H*_O_) inferred for all eight sites increased significantly with sample size (Spearman's rank correlation, *ρ* = 0.939, *p* value < 0.001). None of the eight sites showed an excess of heterozygotes (all *p* values < 0.05) and only NP3, CBF and SA had an excess in homozygotes. Deviations from Hardy–Weinberg equilibrium were significant for NP3 and CBF (Global Hardy–Weinberg score *U*‐test, *p* < 0.01, Table 2) with *H*_O_ values being smaller than the expected heterozygosity (*H*_E_, Table 2). *F*_IS_ values indicating deviations from panmixia were positive and significant for SA (*F*_IS_ = 0.185, Table 2) and NP3 (*F*_IS_ = 0.095, Table 2). The highest mean pairwise relatedness value was found for MV (*r* = 0.281, Table 2), followed by SBF (*r* = 0.184, Table 2), NBF (*r* = 0.134, Table 2), and NP1 (*r* = 0.092, Table 2). NP2 had the lowest relatedness (*r* = 0.051, Table 2). Relatedness values inferred for sites did not correlate with their sample size (Spearman's rank correlation, *ρ* = −0.458, *p* value = 0.254).

### 3.3 STRUCTURE Analysis and Sib Ship Analysis in COLONY

**FIGURE 3:**
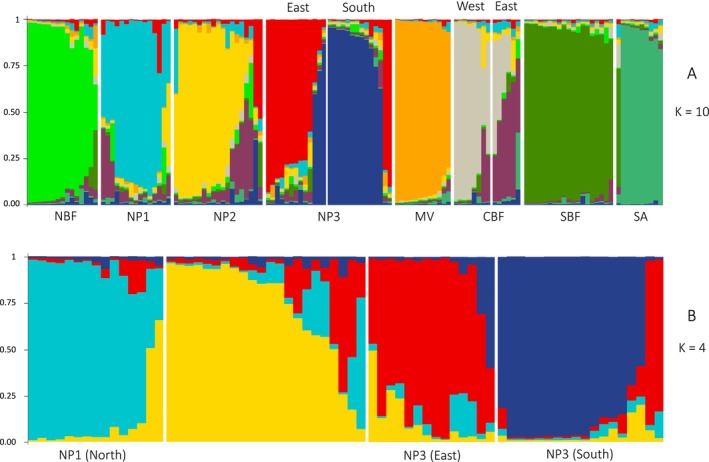
Results of STRUCTURE analysis of *Vipera berus* individuals sampled. Proportional membership coefficients of individuals for the inferred genetic groups are shown. (A) For all individuals of the eight sites (*N* = 131). (B) Only for individuals from NP1, NP2, and NP3 complemented by eight individuals sampled in their vicinity (*N* = 69). Individuals are sorted according to their sampling site and are separated by roads (see Figure 4A).

When analyzing all 131 individuals from the eight sites together, STRUCTURE suggested 10 genetic groups (K) based on the criterion of Evanno et al. (2005), with individuals from NP3 and CBF each comprising two groups (Figure 3). For NP3, STRUCTURE assigned five of all 27 individuals sampled to the opposite side of the road. Only three of 14 individuals sampled at CBF were assigned to this site. As NP3 is crossed by a road and CBF is transected by a small village, we assigned all individuals of NP3 and CBF, respectively, to two subgroups based on whether they were found in the east or south of the road (NP3), or in the east or west of the village (CBF). The resulting four subgroups (and sample sizes) were NP3‐E (*N* = 13) and NP3‐S (*N* = 14) as well as CBF‐E (*N* = 6) and CBF‐W (*N* = 8). Results on Hardy–Weinberg equilibrium and values on *N*_E_ *A*, *A*_R_, *A*_P_, *H*_O_, and *H*_E_ as well as on *F*_IS_ and *r* (each with 95% confidence intervals) are given in Table 2 for each of the four subgroups. For NP3 and CBF, deviations from Hardy–Weinberg equilibrium only disappeared in one of the two subgroups. For both sampling sites, *H*_O_, *H*_E_, and *r* values of subgroups differed significantly, whereas *A*_R_ values of subgroups did not. *N*_E_ *A* only differed significantly between the two subgroups of CBF. *A*_P_ and *F*_IS_ values differed significantly between the subgroups of both CBF and NP3.

When conducting STRUCTURE analysis only with individuals from the sites NP1, NP2 and NP3 and the four individuals sampled in‐between NP1 and NP2 as well as with the four individuals sampled in a village in the south of NP3 (*N* = 63), the optimal number of genetic groups inferred was four. Individuals sampled at NP1 and NP2 were corroborated as distinct groups and those sampled at NP3 were split again into two distinct genetic groups (Figure 3).

**FIGURE 4:**
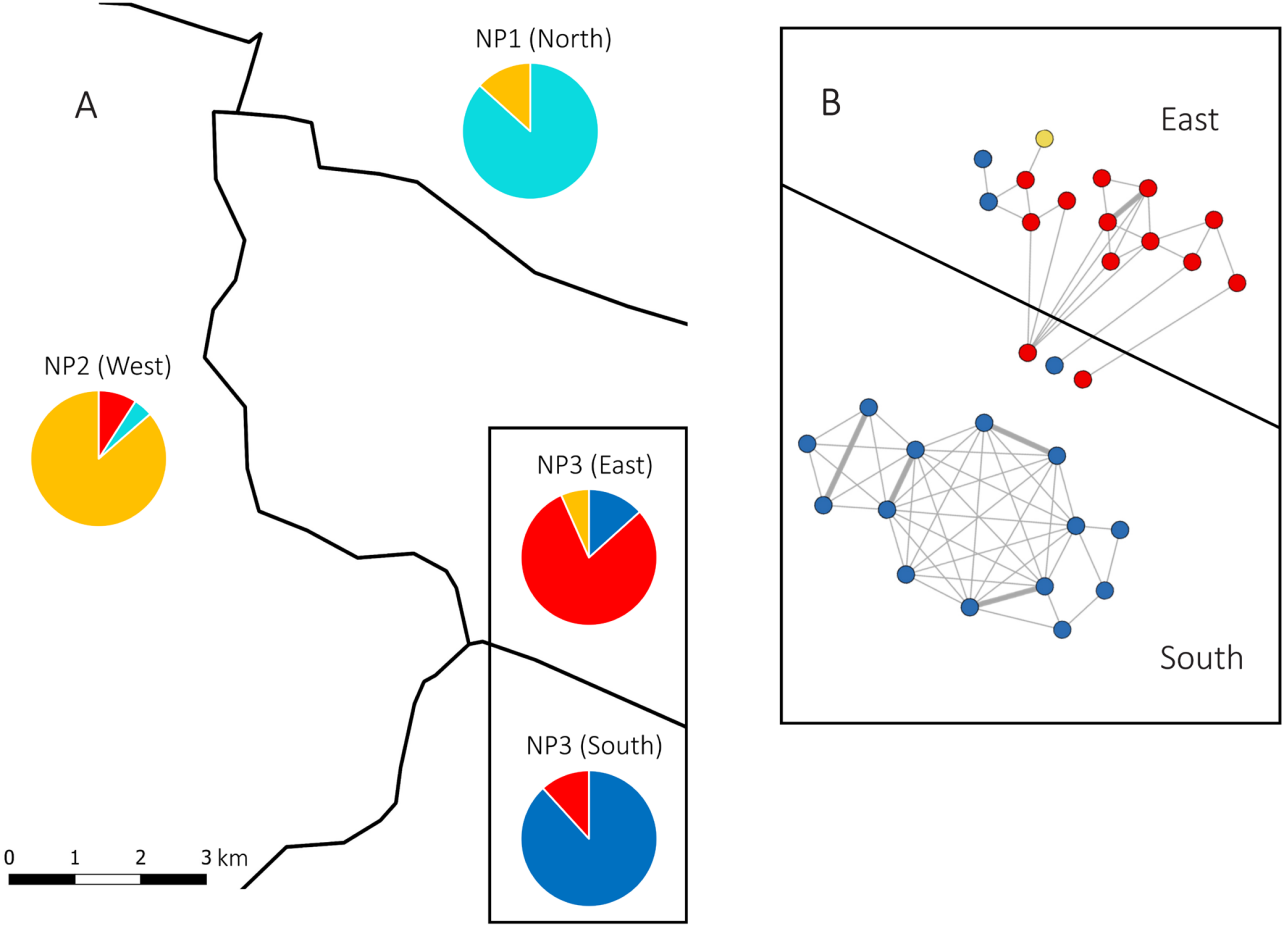
(A) Pie charts on group memberships of individuals of NP1, NP2, NP3 (East, NP3‐E), and NP3 (South, NP3‐E) plotted on a simplified map showing the geographical distribution of sites. Black lines represent roads. Pie charts show for each of the individuals the genetic group for which its membership coefficient was the largest in our STRUCTURE analysis and colors used for genetic groups are as in the STRUCTURE plot (Figure 3B). (B) Network of related individuals for NP3 calculated by COLONY. Half‐sib dyads are given by thin lines and full‐sib dyads by thick lines.

**FIGURE A1:**
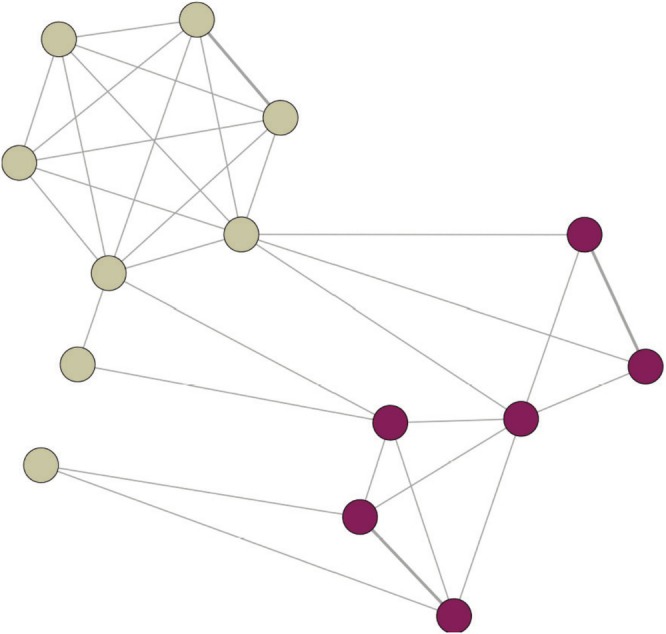
Network on related individuals of CBF. It was derived from COLONY. Half‐sib dyads are given by thin lines and full‐sib dyads by thick lines. CBF‐W: Gray, CBF‐E: Violet. Colors used for individuals are as in STRUCTURE analysis plots (Figure 3A).

**FIGURE A2:**
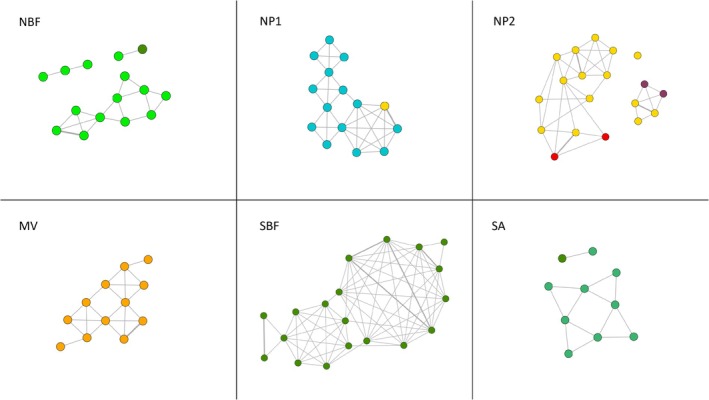
Networks on related individuals of NBF, NP1, NP2, MV, SBF, and SA derived from COLONY. Half‐sib dyads are given by thin lines and full‐sib dyads by thick lines. Colors used for individuals are as in STRUCTURE analysis plots (Figure 3A).

Sibship analysis carried out with the software COLONY corroborated inbreeding for all eight sites. It calculated 64 half sib and five full sib dyads for NP3 (Figure 4), and it suggested 29 half sib and three full sib dyads for CBF (Figure A1 in Appendix). For NP3, COLONY further indicated two fully distinct genetic networks, whereas for CBF no within‐grouping of dyads was apparent (Figure A1 in Appendix). The two networks found for NP3 match the NP3‐E and NP3‐S subgroups suggested by STRUCTURE, except for three individuals of the NP3‐E subgroup that were assigned to the southern side of the road (NP3‐S, Figure 4B). Except for the site SA, for which no full sib dyads were identified, COLONY suggested full sib dyads for all other sites, that is, for NBF (one full sib dyad), NP1 (1), NP2 (3), MV (1), and SBF (5) (Figure A2 in Appendix). Conversely to full sib dyads, half sib dyads were frequent at all eight sites (NBF: 18, NP1: 33, NP2: 35, MV: 20, SBF: 63, SA: 13; Figure A2 in Appendix). For NBF, NP2 and SA, several groups of individuals with first‐degree relationships were suggested (Figure A2 in Appendix).

### 3.4 BOTTLENECK Analysis

Except for MV, for none of our eight sites all four approaches implemented in BOTTLENECK (three tests on whether a population exhibits a significant number of loci with heterozygosity excess and a mode‐shift in allele frequency distributions; Piry et al. 1999) corroborated a past reduction in the effective population size. The “sign test” was significant for NBF (*p* = 0.017), NP2 (*p* = 0.025), NP3 (*p* = 0.004) and MV (*p* = 0.008) and was marginal significant for NP1 (*p* = 0.086) and CBF (*p* = 0.054). The Wilcoxon two tails test on heterozygosity excess and deficiency (“standardized differences test”) was significant for NP1 (*p* = 0.010), NP2 (*p* = 0.002), NP3 (*p* = 0.002) and MV (*p* = 0.014) and marginal significant for NBF (*p* = 0.084). For the latter five sites, “Wilcoxon signed‐rank tests” on heterozygosity excess were also significant (NBF: *p* = 0.042, NP1: *p* = 0.005, NP2: *p* = 0.001, NP3: *p* = 0.001, MV: *p* = 0.007). “Mode‐shifts” indicating no stable populations were evidenced for MV and CBF.

### 3.5 Genetic Differentiation Between Sites

All pairwise *F*_ST_ values of the eight sites calculated across the 10 loci differed significantly from zero (Table 3). On average, pairwise *F*_ST_ values were highest for MV (mean *F*_ST_ = 0.275, Table 3) and SBF (mean *F*_ST_ = 0.247, Table 3) with MV showing the second smallest average geographic distance to all other sites (29.7 km) and SBF the largest (72.7 km, Table 3). Isolation by distance (IBD) was not significant when analyzing all eight sites together (Mantel test, *r* ^2^ = 0.621, *p* value = 0.505). When excluding MV, IBD was significant (Mantel test, *r* ^2^ = 0.827, *p* value = 0.015), whereas when excluding SBF it was not (Mantel test, *r* ^2^ = 0.031, *p* value = 0.560). When excluding MV and SBF, IBD was not significant again (Mantel test, *r* ^2^ = 0.3, *p* value = 0.211). Numbers of private alleles observed at sites did not correlate to average pairwise *F*_ST_ values (Spearman's rank correlation, *ρ* = −0.124, *p* value = 0.771; Tables 2 and 3) even when MVP was excluded (Spearman's rank correlation, *ρ* = 0.112, *p* value = 0.811).

**TABLE 3:** Pairwise *F*_ST_ values (above diagonal) inferred from the analysis of 10 microsatellite loci and geographic distances [km] (below diagonal) of eight sites at which *Vipera berus* individuals were sampled as well as the mean *F*_ST_ value for each site.

	NBF	NP1	NP2	NP3	MV	CBF	SBF	SA	∅
NBF	—	0.164	0.169	0.154	0.313	0.182	0.276	0.226	0.212
NP1	20.0	—	0.092	0.085	0.311	0.101	0.266	0.136	0.166
NP2	24.5	4.0	—	0.076	0.242	0.080	0.216	0.146	0.146
NP3	28.0	11.0	8.0	—	0.274	0.131	0.232	0.184	0.162
MV	27.0	16.5	15.5	9.0	—	0.277	0.413	0.370	0.314
CBF	47.0	31.0	28.0	20.5	20.5	—	0.293	0.211	0.182
SBF	95.5	78.5	73.5	68.5	68.5	48.0	—	0.276	0.282
SA	65.5	67.0	66.5	59.5	51.0	53.0	76.5	—	0.221
∅	43.9	32.6	31.4	29.2	29.7	35.4	72.7	62.7	—

*Note:* All pairwise *F*_ST_ values differ significantly from zero.

## 4 Discussion

In our study, we assessed genetic diversity and within and between genetic differentiation of adders sampled at eight sites in Baden‐Württemberg (BW) to infer whether and how a decline in population sizes and in the number of adder populations could have affected the adder's genetic constitution in this region.

### 4.1 Genetic Diversity and Bottlenecks

We expected small populations (i.e., sites of small sample sizes) to show a lower genetic diversity and higher relatedness than large populations. Conversely, we found no correlation of allelic richness (*A*_R_) and relatedness (*r*), respectively, with sample size. Larger historic population sizes might have led to higher *A*_R_ values than expected from the current number of individuals living at a site and vice versa. At the four sites at which relatedness was highest (MV, SBF, NBF, SA), individuals were sampled at linear structures, such as rock walls, forest edges, and embankments, whereas at three of the four sites with the lowest relatedness values (NP1, NP2, NP3), areas were broad and individuals had a more random spatial distribution. In linear habitats, distances between individuals are generally smaller than in broader habitats just as distances of philopatric individuals to their reproductive sites. Our findings are consistent with studies reporting that high philopatry and low vagility ultimately lead to a high relatedness among individuals in small populations (Duthie et al. 2016; Shields 1982). Observed heterozygosity (*H*_O_) at our eight sampling sites increased significantly with increasing sample size and corroborated an increasing genetic diversity with increasing sample size, although NP3, CBF, and SA showed an excess in homozygotes and *F*_IS_ was significant for NP3 (*F*_IS_ = 0.095) and SA (*F*_IS_ = 0.185). Similar to our results, in a small and inbred adder population in Sweden, heterozygosity was lower and genetic similarity was higher than in larger and less inbred populations studied by the authors (Madsen et al. 1996, 1999).

Allelic richness (*A*_R_), observed (*H*_O_) and expected heterozygosity (*H*_E_) of our eight sites sampled fit well to values observed in microsatellite studies conducted on the adder in other European regions (Table 4). Nevertheless, their variability in genetic diversity stands out (Table 4). Their broad ranges question the central‐marginal hypothesis on postglacial recolonization of the Jura mountains (Ursenbacher et al. 2015). This hypothesis predicts a decrease in genetic diversity with an increasing distance from the refugium and thus would imply that all, and not only a few, adder populations in BW have low genetic diversities compared to populations located closer to the refugium. The large ranges seen in the three measures of genetic diversity further reject that a historic event at a large geographic scale had reduced simultaneously the genetic diversity of the adder throughout BW. Conversely, they may indicate that local factors, such as bottlenecks and founder effects, had affected and still affect the genetic diversity of adders at our eight sites.

**TABLE 4:** Summary statistics of several microsatellite studies conducted in Europe on *Vipera berus* .

References	Region	#Pops	*N*	*A*_R_	*H*_O_	*H*_E_	*F*_ST_
This study	BW	8	6–27	2.533–4.147	0.467–0.653	0.444–0.616	0.080–0.413
Ursenbacher et al. (2009)	Jura Mountains	10	12–63 (34^a^)	2.190–3.140	0.476–0.560	0.387–0.589	0.041–0.415
Ursenbacher et al. (2009)	Alps	3	20–23	2.11–3.000	0.347–0.638	0.376–0.589	0.315–0.417
Ursenbacher et al. (2009)	Massif Central	2	5–18	3.860–4.040	0.556–0.629	0.679–0.681	0.069
Ursenbacher et al. (2009)	Rennes, France	1		3.800	0.501	0.650	
Ball et al. (2020)	Great Britain	16	4–25	1.990–2.790	0.460–0.770	0.500–0.760	0.100–0.164^b^

Abbreviations: *A*_R_ = range of allelic richness; *F*_ST_ = range of significant average pairwise fixation indices; *H*_E_ = range of expected heterozygosity; *H*_O_ = range of observed heterozygosity; *N* = range of sample sizes; Pops = number of populations.

^a^ Without two Swiss populations (*N* = 63, *N* = 62).

^b^ For comparison, only 10 populations with similar geographic distances as in our study are shown.

For SBF (southern Black Forest) and SA (Swabian Jura), there was no evidence on a past bottleneck or founder effect, that is, the software BOTTLENECK detected no mode‐shift and all three tests on heterozygosity excess were not significant. Conversely, a mode‐shift and a heterozygosity excess was indicated by all three tests for MV, which is located in the northern Black Forest. For our other five sites (NBF, NP1, NP2, NP3, CBF) in the northern Black Forest, statistical evidence on bottlenecks and founder effects was ambiguous. As BOTTLENECK requires at least allele frequency data from samples of 20–30 individuals and four polymorphic loci (Piry et al. 1999), this might generally question all these results (Table 2). For the northern Black Forest, however, persecution of adders is documented by town councils of BW (Fritz et al. 2007) and thus, reductions in population sizes in the past. In particular, in the area of the five sites NBF, NP1, NP2, NP3, and MV proven high numbers of adders had been killed in the years 1907–1913 (Fritz et al. 2007). Furthermore, a mode shift and heterozygosity excess inferred by BOTTLENECK analysis can also indicate a sudden reduction in gene flow for the population sample (Broquet et al. 2010; Ryman et al. 2019). A change in forestry in the mid 1900s, leading to overgrowth of many open areas in the entire Black Forest is also documented, which led to shrinkage and fragmentation of adder habitats (Fritz et al. 2007; Ludemann 2015). These changes in forestry not only likely went along with decreases in gene flow between populations but also in population sizes. Thus, our ambiguous statistical evidence on bottlenecks and founder effects for the sites NBF, NP1, NP2, NP3, and CBF would indicate that all these past anthropogenically caused decreases in population size would have not been severe enough to be clearly detected by the BOTTLENECK software. This would be consistent with that several population genetic studies conducted on vipers in other geographic regions also found no bottlenecks (Ferchaud et al. 2011; Simonov and Wink 2012; Ursenbacher et al. 2009).

### 4.2 Genetic Differentiation Within Sites

Due to the past anthropogenic negative influences on adder populations, we expected signs of genetic erosion in adders for the eight sites sampled. The high number of full sib and half sib dyads found by sibship analysis (Figure 4, Figures A1 and A2 in Appendix) and the significantly high average pairwise relatedness values indicate that inbreeding is substantial among adders at all eight sites. Within the two sites NP3 and CBF, which are bisected by a road (NP3) and a small village (CBF), respectively, we found a high differentiation. Differentiation was indicated by a significant *F*_IS_ value (0.095) for NP3, by Hardy–Weinberg disequilibrium for NP3 and CBF (Table 2) and by STRUCTURE analysis yielding two genetic subgroups for the individuals sampled at the NP3 and CBF site, respectively (Figure 3A). For a test of whether this differentiation might reflect an anthropogenic barrier to gene flow (see Ball et al. 2020 and Pozzi et al. 2023 for the adder; Doherty et al. 2020 and Moore et al. 2008 for reptiles), we split individuals sampled at each site into two subgroups according to their finding location relative to the potential barrier. Only for one of the two subgroups of each site did Hardy–Weinberg disequilibria disappear (Table 2). Notably, whereas NP3 had a relatively low, yet significant *F*_IS_ value (0.095) that of subgroup NP3‐E was even higher and significant (*F*_IS_ = 0.213), whereas the nonsignificant *F*_IS_ value of NP3S indicated no inbreeding (Table 2). All this suggests that further factors add to the substantial differentiation within CBF and NP3. For CBF, COLONY analysis suggested 29 half sib and three full sib dyads, but the sibship network on individuals did not corroborate the two subgroups of individuals identified by our STRUCTURE analysis (Figure A1 in Appendix). This contradicts that the village transecting CBF reduced mating opportunities. Conversely, the STRUCTURE analysis conducted with all individuals from the NP1, NP2, and N3 sites and eight individuals sampled in the vicinity of these sites yielded four genetic groups located at different roadsides (Figure 4A) and this spatial pattern was corroborated by COLONY analysis (Figure 4B, Figure A2 in Appendix). Only three individuals found at the NP3‐S roadside were in the NP3‐E sibship cluster (Figure 4B). Remarkably, all these individuals were found at the same hibernaculum, which was located close to the road at the NP3‐S site. The complete absence of half‐sib dyads (Figure 4B) corroborates a high differentiation between NP3S and NP3‐E at a very small spatial scale. A barrier function of roads was also found by Ball et al. (2020) for the adder.

All these results point to a substantial reduction in gene flow among adders within the eight sites and a genetic erosion at sites. However, philopatric mating systems always cause spatial genetic substructures and sibships among individuals (Madsen and Shine 1992a). As we could not run the COLONY algorithm with a complete pedigree or any information on parenthoods of individuals, it is possible that sibships and in particular half sibs inferred likely represent identity by descent (Jones and Wang 2010). This is corroborated by the mean pairwise relatedness values of NP3‐E and NP3W as well as of CBF‐E and CBF‐W, which are larger than that of NP3 and CBF, respectively (Table 2). The high *A*_R_‐value of NP3, NP3‐E and NP3W as well as of CBF, CBF‐E and CBF‐W might question any within‐site effect of anthropogenic structures but could also reflect a conservation of alleles from larger and more connected populations in the past (Pozzi et al. 2023).

### 4.3 Genetic Differentiation Between Sites

Our high pairwise *F*_ST_ values inferred across BW (Figure 2, Table 3) are in the range of values found in other microsatellite studies conducted on adder populations of similar geographic distances (Table 4). Only populations from the Swiss Jura and from the Alps studied by Ursenbacher et al. (2009) show a similar range of pairwise *F*_ST_ values. Conversely, pairwise *F*_ST_ values of two populations in the Massif Central that were also investigated by Ursenbacher et al. (2009) were even smaller than the smallest value observed by us and the values that Ball et al. (2020) inferred from 16 populations in Great Britain were intermediate to that of this study. All this indicates that genetic differentiation between sites is much higher in BW, the Jura Mountains and the Alps than in the Massif Central and Great Britain. This is most probably due to striking differences in the topography of the three former and the two latter geographic regions. The altitudinal heterogeneous topography of BW, the Jura Mountains and the Alps hampers or even goes along with natural barriers to gene flow. Consistent with this, we found at least one private allele at all eight sites (Table 2), and that a correlation between geographic distance and pairwise *F*_ST_ values (IBD) and numbers of private alleles, respectively was absent. For example, only samples of the three sites NP1, NP2 and NP3 with the smallest geographical distances to each other out of all eight sites studied (Table 3) had pairwise *F*_ST_ values smaller than 0.100. The pairwise *F*_ST_ value of the NP2 and CBF site (*F*_ST_ = 0.080, geographic dist. = 28 km, Table 3), however, was smaller than that between NP1 and NP2 (*F*_ST_ = 0.092, dist. = 4 km), even though the distance between NP2 and CBF is seven times higher than between NP1 and NP2.

### 4.4 Methodological Limitations

Despite the availability of many genomic approaches today (e.g., SNP genotyping, RAD sequencing), which can assay the genetic diversity of contemporary populations at an unprecedented scale, we chose microsatellites for our study for several reasons. To date, genomic approaches are relatively underutilized when studying reptiles (Hayden Bofill and Blom 2024, see Pozzi et al. 2023 for the adder) and comparable studies on the European adder are lacking. There are multiple well‐established tools for analyzing genetic diversity and within and between genetic differentiation of populations from microsatellite loci, whereas tools available for genomic data can answer a less broad spectrum of population genetic questions. We are aware that microsatellites come along with methodological limitations, which were apparent in our study and might limit the comparability of results between studies, favoring the use of genomic approaches. Current studies differ in sets of loci applied and use primers exclusively developed for the European adder or a mixture of primers developed for *V. aspis* and *V. ursinii* . However, primer sets finally applied in a study are influenced by the population samples genotyped, which in turn hamper their standardization across independent studies. For example, Vb21 was polymorphic in the studies of Ursenbacher et al. (2009) and Ursenbacher et al. (2015), whereas it was monomorphic in ours. Likewise, Vb‐A8 worked in Ursenbacher et al. (2009) and Ursenbacher et al. (2015), whereas null alleles were evident in our study. Conversely, primers developed for *V. aspis* and *V. ursinii* worked in Ball et al. (2020) and herein (but Vu38 turned out to be monomorphic). Studies also differ in allele scoring methods used (authors score manually, apply different software or do a combination of both), although these influence allele numbers of loci and other measures on genetic diversity. Given that modern high‐throughput sequencing is much less expensive than microsatellite analysis and that population genetic tools are rapidly evolving, we anticipate that they will shortly replace microsatellites. For example, poolSeq can reveal the genetic diversity of an entire population in a single sequencing run, which makes monitoring of a population's genetic diversity over multiple generations comparable cheap and standardizable for species conservation.

## 5 Conclusions and Implications for Adder Conservation

In this study, we established the current genetic constitution of adders at six sites in the northern Black Forest, at one site in the southern Black Forest and at one site in the Swabian Jura. Compared to studies that had been conducted in other European regions at similar geographic scales (Table 4), genetic diversities assessed from *A*_R_, *H*_O_ and *H*_E_ were among the highest and lowest values found. We also inferred high average pairwise relatedness values for all sites and high frequencies of sibship relationships between individuals. Statistical evidence on past bottlenecks and founder effects was ambiguous for most sites. For NP3, we could evidence that a road is an anthropogenic barrier to gene flow within the site. High pairwise *F*_ST_ values and the presence of private alleles at all sites suggest limited gene flow between all sites (Table 3).

We interpret the current genetic constitution of the adder in BW as the result of past persecution (Otte et al. 2020) and of past and ongoing habitat fragmentation and loss (Fritz et al. 2007), which all together led to a decrease in genetic diversity and gene flow and an increase in genetic erosion at local or regional scales. Based on our results, we suggest the establishment of corridors and new habitats of high quality at a local and regional scale to increase the chance for long‐term survival of the adder throughout BW. Increased gene flow will lead to an exchange of alleles (private alleles would be novel to other populations) between sites, which is crucial due to the low genetic diversity and the many sibship relationships observed at most of the sites studied. As we have pictures of the head scales of all individuals genotyped, translocation of individuals with genotypes novel to other populations might be an option in the face of global warming to accelerate an overall genetic homogenization of adder populations and increase their fitness. Translocation would also speed up colonization of new habitats (Ball et al. 2024). When inbreeding depression is evident for a population (e.g., Madsen et al. 1996; note that adders showed no inbreeding depression at all our study sites) translocation would also be an option. Our results underline the urgent need for conservation strategies and endorse long‐term monitoring (see Ball et al. 2024) using standardized genetic markers to check the genetic status of the adder in BW. As conservation genetics in *Vipera* continuously evolves, integrating microsatellites with high‐throughput sequencing tools may provide a comprehensive understanding of population structure and resilience of the adder over time in BW.

## Author Contributions

**P. Lennart Schmid:** conceptualization (equal), data curation (equal), formal analysis (equal), investigation (equal), methodology (equal), validation (equal), visualization (equal), writing – original draft (equal), writing – review and editing (equal). **Judith Grünewald:** conceptualization (equal), data curation (equal), formal analysis (equal), investigation (equal), methodology (equal), validation (equal), visualization (equal), writing – original draft (equal), writing – review and editing (equal). **Marc I. Förschler:** conceptualization (supporting), funding acquisition (lead), resources (supporting), supervision (supporting), writing – review and editing (supporting). **Eva Maria Griebeler:** conceptualization (equal), funding acquisition (supporting), investigation (supporting), methodology (supporting), project administration (lead), resources (lead), supervision (lead), validation (equal), writing – review and editing (equal).

## Conflicts of Interest

The authors declare no conflicts of interest.

## Data Availability

All data used in the paper are listed in the paper and in Appendix.

## Appendix

**TABLE A1:** DNA yield per swap.

Site	*N*	Mean
NBF	15	38.16
NP1	15	16.48
NP2	20	35.67
NP3	29	50.05
MV	12	31.90
CBF	14	22.44
SBF	19	31.72
SA	10	39.42
All	142	38.24

*Note:* Average DNA yield is given in ng/μL for each site and all adder individuals sampled (134 sampled at all sites plus four in‐between the sites NP1 and NP2 and four others in a village south of NP3).

## References

[ece371644-bib-0001] Alford, R. A. , and S. J. Richards . 1999. “Global Amphibian Declines: A Problem in Applied Ecology.” Annual Review of Ecology and Systematics 30, no. 1: 133–165. 10.1146/annurev.ecolsys.30.1.133.

[ece371644-bib-0002] Arif, I. A. , H. A. Khan , M. Shobrak , et al. 2010. “Interpretation of Electrophoretograms of Seven Microsatellite Loci to Determine the Genetic Diversity of the Arabian Oryx.” Genetics and Molecular Research 9, no. 1: 259–265. 20198581 10.4238/vol9-1gmr714

[ece371644-bib-0003] Aßmann, T. , and P. Finck . 2020. “Rote‐Liste‐Gremium Amphibien und Reptilien. Rote Liste und Gesamtartenliste der Reptilien (Reptilia) Deutschlands.” Naturschutz und Biologische Vielfalt 170, no. 3: 64.

[ece371644-bib-0004] Ball, S. , N. Hand , F. Willman , et al. 2020. “Genetic and Demographic Vulnerability of Adder Populations: Results of a Genetic Study in Mainland Britain.” PLoS One 15, no. 4: e0231809. 32310990 10.1371/journal.pone.0231809 PMC7170227

[ece371644-bib-0005] Ball, S. , S. Petrovan , E. Ashe‐Jepson , et al. 2024. “Genetic Study of an Isolated Population of Adders *Vipera berus* Founded by Historic Translocation: Implications for Conservation.” Herpetological Journal 34: 197–210.

[ece371644-bib-0006] Bauwens, D. , and K. Claus . 2019. “Seasonal Variation of Mortality, Detectability, and Body Condition in a Population of the Adder ( *Vipera berus* ).” Ecology and Evolution 9, no. 10: 5821–5834. 31161001 10.1002/ece3.5166 PMC6540836

[ece371644-bib-0007] Bauwens, D. , K. Claus , and J. Mergeay . 2018. “Genotyping Validates Photo‐Identification by the Head Scale Pattern in a Large Population of the European Adder ( *Vipera berus* ).” Ecology and Evolution 8, no. 5: 2985–2992. 29531711 10.1002/ece3.3917 PMC5838086

[ece371644-bib-0008] Broquet, T. , S. Angelone , J. Jaquiéry , et al. 2010. “Genetic Bottlenecks Driven by Population Disconnection.” Conservation Biology 24, no. 6: 1596–1605. 20666803 10.1111/j.1523-1739.2010.01556.x

[ece371644-bib-0009] Carlsson, M. , M. Isaksson , M. Höggren , and H. Tegelström . 2003. “Characterization of Polymorphic Microsatellite Markers in the Adder, *Vipera berus* .” Molecular Ecology Notes 3, no. 1: 73–75. 10.1046/j.1471-8286.2003.00354.x.

[ece371644-bib-0010] Cornuet, J. M. , and G. Luikart . 1996. “Description and Power Analysis of Two Tests for Detecting Recent Population Bottlenecks From Allele Frequency Data.” Genetics 144, no. 4: 2001–2014. 10.1093/genetics/144.4.2001. 8978083 PMC1207747

[ece371644-bib-0011] Doherty, T. S. , S. Balouch , K. Bell , et al. 2020. “Reptile Responses to Anthropogenic Habitat Modification: A Global Meta‐Analysis.” Global Ecology and Biogeography 29, no. 7: 1265–1279. 10.1111/geb.13091.

[ece371644-bib-0012] Duthie, A. B. , G. Bocedi , and J. M. Reid . 2016. “When Does Female Multiple Mating Evolve to Adjust Inbreeding? Effects of Inbreeding Depression, Direct Costs, Mating Constraints, and Polyandry as a Threshold Trait.” Evolution 70, no. 9: 1927–1943. 10.1111/evo.13005. 27464756 PMC5053304

[ece371644-bib-0013] Evanno, G. , S. Regnaut , and J. Goudet . 2005. “Detecting the Number of Clusters of Individuals Using the Software STRUCTURE: A Simulation Study.” Molecular Ecology 14, no. 8: 2611–2620. 10.1111/j.1365-294X.2005.02553.x. 15969739

[ece371644-bib-0014] Ferchaud, A. L. , A. Lyet , M. Cheylan , et al. 2011. “High Genetic Differentiation Among French Populations of the Orsini's Viper ( *Vipera ursinii ursinii* ) Based on Mitochondrial and Microsatellite Data: Implications for Conservation Management.” Journal of Heredity 102, no. 1: 67–78. 10.1093/jhered/esq098. 20841316

[ece371644-bib-0015] François, D. , S. Ursenbacher , A. Boissinot , F. Ysnel , and O. Lourdais . 2021. “Isolation by Distance and Male Biased Dispersal at a Fine Spatial Scale: A Study of the Common European Adder (*Vipera berus*) in a Rural Landscape.” Conservation Genetics 22: 823–837.

[ece371644-bib-0016] Frankham, R. 1996. “Relationship of Genetic Variation to Population Size in Wildlife.” Conservation Biology 10, no. 6: 1500–1508. 10.1046/j.1523-1739.1996.10061500.x.

[ece371644-bib-0017] Fritz, K. , M. Lehnert , and P. Sowig . 2007. “Kreuzotter. *Vipera berus* (Linnaeus, 1758).” In Die Amphibien und Reptilien Baden‐Württembergs, edited by H. Laufer , K. Fritz , and P. Sowig , 709–732. Ulmer.

[ece371644-bib-0018] Gardner, E. , A. Julian , C. Monk , and C. Baker . 2019. “Make the Adder Count: Population Trends From a Citizen Science Survey of UK Adders.” Herpetological Journal 29: 57–70. 10.13140/RG.2.2.18815.43682.

[ece371644-bib-0019] Gardner, T. A. , J. Barlow , and C. A. Peres . 2007. “Paradox, Presumption and Pitfalls in Conservation Biology: The Importance of Habitat Change for Amphibians and Reptiles.” Biological Conservation 138, no. 1–2: 166–179. 10.1016/j.biocon.2007.04.017.

[ece371644-bib-0020] Geser, S. , L. Kaiser , V. Zwahlen , and S. Ursenbacher . 2013. “Development of Polymorphic Microsatellite Loci Markers for the Asp Viper (*Vipera aspis*) Using High‐Throughput Sequencing and Their Use for Other European Vipers.” Amphibia‐Reptilia 34, no. 1: 109–113. 10.1163/15685381-00002861.

[ece371644-bib-0021] Gibbons, J. W. , D. E. Scott , T. J. Ryan , et al. 2000. “The Global Decline of Reptiles, Déjà vu Amphibians.” Bioscience 50, no. 8: 653–666. 10.1641/0006-3568(2000)050[0653:TGDORD]2.0.CO;2.

[ece371644-bib-0022] Goudet, J. 1995. “FSTAT (Version 1.2): A Computer Program to Calculate F‐Statistics.” Journal of Heredity 86, no. 6: 485–486. 10.1093/oxfordjournals.jhered.a111627.

[ece371644-bib-0023] Guillon, M. 2012. “De la Physiologie à la Répartition: Adaptations Climatiques et Sensibilité Thermique Chez une Relique Glaciaire.” Sciences de l'Environnement. Faculté des Sciences Fondamentales et Appliquées—Université de Poitiers, Français. https://theses.hal.science/tel‐00996071v1.

[ece371644-bib-0024] Hayden Bofill, S. I. , and M. P. K. Blom . 2024. “Climate Change From an Ectothermic Perspective: Evolutionary Consequences and Demographic Change in Amphibian and Reptilian Populations.” Biodiversity and Conservation 33: 905–927.

[ece371644-bib-0025] IUCN . 2025. “The IUCN Red List of Threatened Species. Version 2024‐2.” https://www.iucnredlist.org.

[ece371644-bib-0026] Jones, O. R. , and J. Wang . 2010. “COLONY: A Program for Parentage and Sibship Inference From Multilocus Genotype Data.” Molecular Ecology Resources 10, no. 3: 551–555. 10.1111/j.1755-0998.2009.02787.x. 21565056

[ece371644-bib-0027] Laufer, H. , K. Fritz , and P. Sowig . 2007. Die Amphibien und Reptilien Baden‐Württembergs, 808. Ulmer.

[ece371644-bib-0028] Linnaeus, C. 1758. “Description of *Coluber berus* (= *Vipera berus*).” Systema Naturae per Regna Tria Naturae, Secundum Classes, Ordines, Genera, Species, Cum Characteribus, Differentiis, Synonymis, Locis. Tomus I. Editio Decima, Reformata. Laurentii Salvii, Holmiae, 217.

[ece371644-bib-0029] Ludemann, T. 2015. “History, Landscape and Vegetation in the Black Forest, SW Germany.” Tuexenia Beihefte 6: 29–85.

[ece371644-bib-0030] Luiselli, L. 1993. “High Philopatry Can Produce Strong Sexual Competition in Male Adders, *Vipera berus* .” Amphibia‐Reptilia 4: 310–311.

[ece371644-bib-0031] Lynch, M. , and K. Ritland . 1999. “Estimation of Pairwise Relatedness With Molecular Markers.” Genetics 152, no. 4: 1753–1766. 10.1093/genetics/152.4.1753. 10430599 PMC1460714

[ece371644-bib-0032] Madsen, T. , and R. Shine . 1992a. “Determinants of Reproductive Success in Female Adders, *Vipera berus* .” Oecologia 92, no. 1: 40–47. 10.1007/BF00317260. 28311810

[ece371644-bib-0033] Madsen, T. , and R. Shine . 1992b. “Sexual Competition Among Brothers May Influence Offspring Sex Ratio in Snakes.” Evolution 46, no. 5: 1549–1552. 28568982 10.1111/j.1558-5646.1992.tb01144.x

[ece371644-bib-0034] Madsen, T. , R. Shine , M. Olsson , and H. Wittzel . 1999. “Restoration of an Inbred Adder Population.” Nature 402: 34–35.

[ece371644-bib-0035] Madsen, T. , B. Stille , and R. Shine . 1996. “Inbreeding Depression in an Isolated Population of Adders *Vipera berus* .” Biological Conservation 75, no. 2: 113–118. 10.1016/0006-3207(95)00067-4.

[ece371644-bib-0036] Metzger, C. , A. L. Ferchaud , C. Geiser , and S. Ursenbacher . 2011. “New Polymorphic Microsatellite Markers of the Endangered Meadow Viper ( *Vipera ursinii* ) Identified by 454 High‐Throughput Sequencing: When Innovation Meets Conservation.” Conservation Genetics Resources 3, no. 3: 589–592. 10.1007/s12686-011-9411-x.

[ece371644-bib-0037] Moore, J. A. , H. C. Miller , C. H. Daugherty , and N. J. Nelson . 2008. “Fine‐Scale Genetic Structure of a Long‐Lived Reptile Reflects Recent Habitat Modification.” Molecular Ecology 17, no. 21: 4630–4641. 10.1111/j.1365-294X.2008.03951.x. 19140986

[ece371644-bib-0038] Otte, N. , D. Bohle , and B. Thiesmeier . 2020. Die Kreuzotter. Ein Leben in Ziemlich Festen Bahnen. Laurenti (Beiheft der Zeitschrift für Feldherpetologie).

[ece371644-bib-0039] Peakall, R. , and P. E. Smouse . 2012. “GenAlEx 6.5: Genetic Analysis in Excel. Population Genetic Software for Teaching and Research—An Update.” Bioinformatics 28, no. 19: 2537–2539. 10.1093/bioinformatics/bts460. 22820204 PMC3463245

[ece371644-bib-0040] Piry, S. , G. Luikart , and J. M. Cornuet . 1999. “BOTTLENECK: A Computer Program for Detecting Recent Reductions in the Effective Size Using Allele Frequency Data.” Journal of Heredity 90, no. 4: 502–503. 10.1093/jhered/90.4.502.

[ece371644-bib-0041] Pozzi, A. V. , J. B. Ownes , B. Üveges , et al. 2023. “High Standing Diversity Masks Extreme Genetic Erosion in a Declining Snake.” *bioRxiv* 2023.09.19.557540. 10.1101/2023.09.19.557540.

[ece371644-bib-0042] Pritchard, J. K. , M. Stephens , and P. Donnelly . 2000. “Inference of Population Structure Using Multilocus Genotype Data.” Genetics 155, no. 2: 945–959. 10.1093/genetics/155.2.945. 10835412 PMC1461096

[ece371644-bib-0043] Raymond, M. , and F. Rousset . 1995. “GENEPOP (Version 1.2): Population Genetics Software for Exact Tests and Ecumenicism.” Journal of Heredity 86, no. 3: 248–249. 10.1093/oxfordjournals.jhered.a111573.

[ece371644-bib-0044] Ryman, N. , L. Laikre , and O. Hössjer . 2019. “Do Estimates of Contemporary Effective Population Size Tell Us What We Want to Know?” Molecular Ecology 28, no. 8: 1904–1918. 10.1111/mec.15027. 30663828 PMC6850010

[ece371644-bib-0045] Schiemenz, H. 1985. Die Kreuzotter. Vipera berus. Neue Brehm‐Bücherei.

[ece371644-bib-0046] Shields, W. M. 1982. Philopatry, Inbreeding, and the Evolution of Sex. State University of New York Press.

[ece371644-bib-0047] Simonov, E. , and M. Wink . 2012. “Population Genetics of the Halys Pit Viper ( *Gloydius halys* ) at the Northern Distribution Limit in Siberia.” Amphibia‐Reptilia 33, no. 2: 273–283. 10.1163/156853812X642045.

[ece371644-bib-0048] Tingley, R. , S. Meiri , and D. G. Chapple . 2016. “Addressing Knowledge Gaps in Reptile Conservation.” Biological Conservation 204: 1–5. 10.1016/j.biocon.2016.07.021.

[ece371644-bib-0049] Ursenbacher, S. , M. Guillon , H. Cubizolle , A. Dupoué , G. Blouin‐Demers , and O. Lourdais . 2015. “Postglacial Recolonization in a Cold Climate Specialist in Western Europe: Patterns of Genetic Diversity in the Adder ( *Vipera berus* ) Support the Central‐Marginal Hypothesis.” Molecular Ecology 24, no. 14: 3639–3651. 10.1111/mec.13259. 26053307

[ece371644-bib-0050] Ursenbacher, S. , J.‐C. Monney , and L. Fumagalli . 2009. “Limited Genetic Diversity and High Differentiation Among the Remnant Adder ( *Vipera berus* ) Populations in the Swiss and French Jura Mountains.” Conservation Genetics 10: 303–315.

[ece371644-bib-0051] van Oosterhout, C. , W. F. Hutchinson , D. P. M. Wills , and P. Shipley . 2004. “Micro‐Checker: Software for Identifying and Correcting Genotyping Errors in Microsatellite Data.” Molecular Ecology Notes 4, no. 3: 535–538. 10.1111/j.1471-8286.2004.00684.x.

[ece371644-bib-0052] Völkl, W. , and B. Thiesmeier . 2002. Die Kreuzotter. Ein Leben in Festen Bahnen? Laurenti.

[ece371644-bib-0053] Wright, S. 1943. “Isolation by Distance.” Genetics 28, no. 2: 114–138. 10.1093/genetics/28.2.114. 17247074 PMC1209196

